# Supplementary material for: Bacillus Calmette-Guérin (BCG) therapy lowers the incidence of Alzheimer’s disease in bladder cancer patients
Source: PLoS One. 2019 Nov 7;14(11):e0224433. doi: 10.1371/journal.pone.0224433 (PMC6837488; doi:10.1371/journal.pone.0224433)
Supplement: S1 Table — (DOCX) [file pone.0224433.s001.docx]

| Age group | Not Given BCG | AD within | AD % | Given BCG | AD within | AD % | TOTAL |
| --- | --- | --- | --- | --- | --- | --- | --- |
| 0-64 | 77 | 0 | 0% | 83 | 0 | 0% | 160 |
| 65-69 | 29 | 0 | 0% | 76 | 0 | 0% | 105 |
| 70-74 | 95 | 3 | 3.16% | 133 | 1 | 0.75% | 228 |
| 75-79 | 74 | 4 | 5.41% | 144 | 5 | 3.47% | 218 |
| 80-84 | 93 | 18 | 19.35% | 163 | 3 | 1.84% | 256 |
| 85-89 | 76 | 11 | 14.47% | 148 | 7 | 4.73% | 224 |
| 90 + | 49 | 8 | 16.33% | 131 | 5 | 3.82% | 180 |
| Total | 493 | 44 | 8.92% | 878 | 21 | 2.39% | 1371 |

**S1 Table. AD and Age distribution of patients (Male and Female**) **not treated or treated with BCG**
