# Supplementary material for: Bacillus Calmette-Guérin (BCG) therapy lowers the incidence of Alzheimer’s disease in bladder cancer patients
Source: PLoS One. 2019 Nov 7;14(11):e0224433. doi: 10.1371/journal.pone.0224433 (PMC6837488; doi:10.1371/journal.pone.0224433)
Supplement: S2 Table — (DOCX) [file pone.0224433.s002.docx]

**S2 Table. AD and Age distribution of patients (Male** **only**) **not treated or treated with BCG**

| Age group | Not Given BCG | AD within | AD % | Given BCG | AD within | AD % | TOTAL |
| --- | --- | --- | --- | --- | --- | --- | --- |
| 0-64 | 63 | 0 | 0% | 68 | 0 | 0% | 131 |
| 65-69 | 24 | 0 | 0% | 61 | 0 | 0% | 85 |
| 70-74 | 77 | 3 | 3.90% | 110 | 1 | 0.91% | 187 |
| 75-79 | 59 | 3 | 5.08% | 133 | 4 | 3.01% | 192 |
| 80-84 | 82 | 16 | 19.75% | 133 | 2 | 1.50% | 215 |
| 85-89 | 60 | 8 | 13.33% | 124 | 7 | 5.65% | 184 |
| 90 + | 39 | 7 | 17.95% | 101 | 4 | 3.96% | 140 |
| Total | 404 | 37 | 9.16% | 730 | 18 | 2.47% | 1134 |
