# Supplementary material for: Bacillus Calmette-Guérin (BCG) therapy lowers the incidence of Alzheimer’s disease in bladder cancer patients
Source: PLoS One. 2019 Nov 7;14(11):e0224433. doi: 10.1371/journal.pone.0224433 (PMC6837488; doi:10.1371/journal.pone.0224433)
Supplement: S3 Table — (DOCX) [file pone.0224433.s003.docx]

| Age group | Not Given BCG | AD within | AD % | Given BCG | AD within | AD % | TOTAL |
| --- | --- | --- | --- | --- | --- | --- | --- |
| 0-64 | 14 | 0 | 0% | 15 | 0 | 0% | 29 |
| 65-69 | 5 | 0 | 0% | 15 | 0 | 0% | 20 |
| 70-74 | 18 | 0 | 0% | 23 | 0 | 0% | 41 |
| 75-79 | 15 | 1 | 6.67% | 11 | 1 | 9.09% | 26 |
| 80-84 | 11 | 2 | 18.18% | 30 | 1 | 3.33% | 41 |
| 85-89 | 16 | 3 | 18.75% | 24 | 0 | 0% | 40 |
| 90 + | 10 | 1 | 10 .00% | 30 | 1 | 3.33% | 40 |
| Total | 89 | 7 | 7.87% | 148 | 3 | 2.03% | 237 |

**S3 Table. AD and Age distribution of patients (Female** **only**) **not treated or treated with BCG**
