# Supplementary material for: Bacillus Calmette-Guérin (BCG) therapy lowers the incidence of Alzheimer’s disease in bladder cancer patients
Source: PLoS One. 2019 Nov 7;14(11):e0224433. doi: 10.1371/journal.pone.0224433 (PMC6837488; doi:10.1371/journal.pone.0224433)
Supplement: S1 Fig — aLog Rank: Chi-Square 6.735, df = 1, p = 0.00945. (DOCX) [file pone.0224433.s004.docx]

**

**

S1 Fig. Kaplan–Meier survival curves of the AD-free female patients according to treatment (BG vs. No BCG) and to age^a^.

^a^Log Rank: Chi-Square 6.735, df=1, p= 0.00945
