# Supplementary material for: Bacillus Calmette-Guérin (BCG) therapy lowers the incidence of Alzheimer’s disease in bladder cancer patients
Source: PLoS One. 2019 Nov 7;14(11):e0224433. doi: 10.1371/journal.pone.0224433 (PMC6837488; doi:10.1371/journal.pone.0224433)
Supplement: S2 Fig — (DOCX) [file pone.0224433.s005.docx]

S2 Fig. Kaplan–Meier survival curves of the AD-free male patients according to treatment (BCG vs. No BCG) and to age^a^.

^a^Log Rank: Chi-Square 35.162, df=1, p= 3.03x10^-9^
