# Supplementary material for: Bacillus Calmette-Guérin (BCG) therapy lowers the incidence of Alzheimer’s disease in bladder cancer patients
Source: PLoS One. 2019 Nov 7;14(11):e0224433. doi: 10.1371/journal.pone.0224433 (PMC6837488; doi:10.1371/journal.pone.0224433)
Supplement: S3 Fig — aLog Rank: Chi-Square 35.162, df = 1, p = 3.03x10^-9^. (DOCX) [file pone.0224433.s006.docx]

Figure S3. Kaplan–Meier survival curves of the AD-free male and female patients not treated with BCG^a^.

^a^Log Rank: Chi-square 0.390, df=1, p=0.533
