# Supplementary material for: Bacillus Calmette-Guérin (BCG) therapy lowers the incidence of Alzheimer’s disease in bladder cancer patients
Source: PLoS One. 2019 Nov 7;14(11):e0224433. doi: 10.1371/journal.pone.0224433 (PMC6837488; doi:10.1371/journal.pone.0224433)
Supplement: S4 Fig — aLog Rank: Chi-square 0.433, df = 1, p = 0.511. (DOCX) [file pone.0224433.s007.docx]

**

**

Figure S1. Kaplan–Meier survival curves of the AD-free female patients according to treatment (BG vs. No BCG) and to age^a^.

^a^Log Rank: Chi-Square 6.735, df=1, p= 0.00945





Figure S4. Kaplan–Meier survival curves of the AD-free male and female patients treated with BCG^a^.

^a^Log Rank: Chi-square 0.433, df=1, p=0.511

**Excel File S1. Variables characterizing the 1371 analyzed patients**
